# Aerosol trace metal leaching and impacts on marine microorganisms

**DOI:** 10.1038/s41467-018-04970-7

**Published:** 2018-07-05

**Authors:** Natalie M. Mahowald, Douglas S. Hamilton, Katherine R. M. Mackey, J. Keith Moore, Alex R. Baker, Rachel A. Scanza, Yan Zhang

**Affiliations:** 1 Department of Earth and Atmospheric Sciences, Atkinson Center for a Sustainable Future, Cornell University, Ithaca, NY USA; 2 Department of Earth System Science, University of California, Irvine, Irvine, CA USA; 3 Centre for Ocean and Atmospheric Sciences, School of Environmental Sciences, University of East Anglia, NR4 7TJ Norwich, UK; 4 Shanghai Key Laboratory of Atmospheric Particle Pollution and Prevention (LAP3), Department of Environmental Science and Engineering, Fudan University, 200433 Shanghai, China; 5 Present Address: Pacific Northwest National Laboratory, Richland, WA, USA

## Abstract

Metal dissolution from atmospheric aerosol deposition to the oceans is important in enhancing and inhibiting phytoplankton growth rates and modifying plankton community structure, thus impacting marine biogeochemistry. Here we review the current state of knowledge on the causes and effects of the leaching of multiple trace metals from natural and anthropogenic aerosols. Aerosol deposition is considered both on short timescales over which phytoplankton respond directly to aerosol metal inputs, as well as longer timescales over which biogeochemical cycles are affected by aerosols.

## Abstract (web summary)

Metal dissolution from atmospheric aerosol deposition plays an important role in enhancing and inhibiting phytoplankton growth and community structure. Here, the authors review the impacts of trace metal leaching from natural and anthropogenic aerosols on marine microorganisms over short and long timescales.

## Introduction

**Fig. 1:**
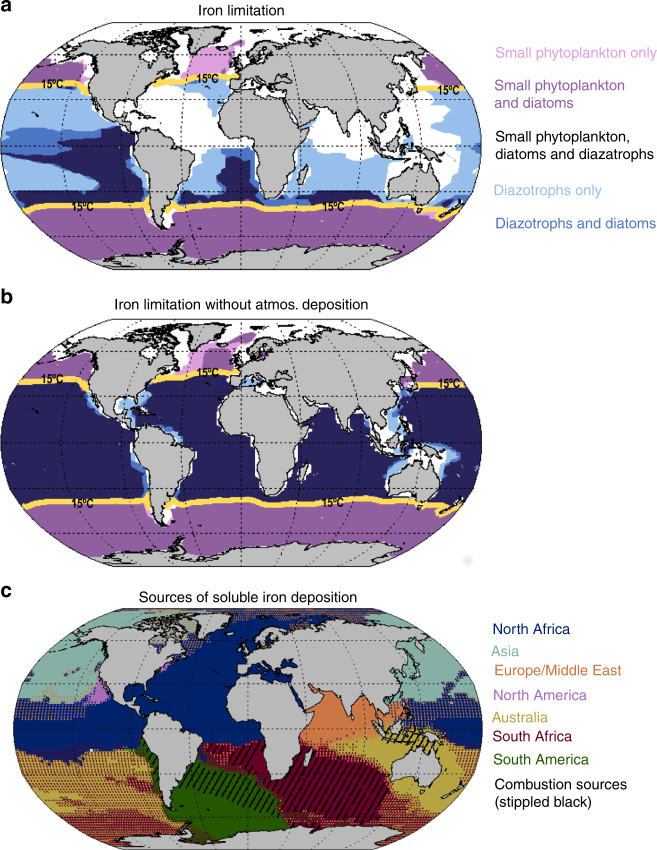
Estimate of iron limitation regions and sources of soluble iron. Estimate of ocean iron limitation regions for diatoms, small phytoplankton, and diazatrophs, based on an ocean biogeochemistry model^161^ (**a**, **b**); iron is limiting for small phytoplankton (light mauve); iron is limiting for diazotrophs and small phytoplankton (purple); iron is limiting for small phytoplankton, diatoms, and diazatrophs (deep purple); iron is limiting for diazatrophs (gray-blue); iron is limiting for diatoms and diazatrophs (blue). This is shown for the current climate (**a**), and in the current climate with no atmospheric deposition of iron (**b**). See Supplementary Figure 1 for more information. Overall, most of the world’s ocean depend on atmospheric iron inputs. Diazotrophs are not present in the model at high latitudes where temperatures are below ~15 °C. The solid yellow line represents the mean location of this temperature control on the diazotrophs. Model-based source apportionment (**c**) for soluble iron deposition into the oceans using R. Scanza et al. (manuscript in preparation) and ref.^160^. The dominant source for each region is identified by color (North Africa: dark blue; East Asia: light green; North America: pink; Australia: yellow; South Africa: red, South America: green). If the soluble iron is contributed substantially from two source regions, the colors are mixed with the dominant source providing the base color, and the secondary source the points. In regions where one source dominates, but the dominant source is not dust, but combustion, black diagonal stippling is applied (in the Southern Hemisphere, near Africa, South America and Micronesia)

The world’s oceans represent a mosaic of diverse but interconnected biogeochemical provinces that cycle elements, support organismal growth and regulate global climate.Marine microorganisms are responsible for approximately half of Earth’s primary productivity, the process that converts carbon dioxide into organic molecules via photosynthesis and chemosynthesis, while at the same time replenishes the oxygen supply. This primary productivity supports the vast biodiversity and fisheries of the ocean. Primary producers like phytoplankton require nutrients to support their growth, and the nutrient in shortest supply relative to cellular requirements limits primary production. Historically, the macronutrients nitrogen (N) and, to a lesser extent phosphorus (P) have been identified as limiting nutrients in the oceans^1,2^. More recently, the role of iron (Fe) in marine biogeochemical cycles as a growth-limiting nutrient for the phytoplankton community in the high nitrate, low chlorophyll (HNLC) regions, located in the Southern Ocean and the equatorial and subarctic Pacific, has been identified (Fig. 1a). Numerous in situ iron fertilization experiments have demonstrated increased growth rates, shifts in community composition, and biomass accumulation with iron addition^3,4^. While other biologically important trace metals do not typically limit productivity to the same extent as iron, as enzyme co-factors they play important roles in determining which enzymes cells can express, modifying microbial community composition and biogeochemistry^5^. Regions where some microbial biogeochemical activity is limited or co-limited by one or more trace metal micronutrients including zinc (Zn), cobalt (Co), and manganese (Mn) have been reported^6–8^. In contrast, some metals, such as copper (Cu) can be toxic at high concentrations to some plankton, and thus additions of some trace metals modifies community composition and inhibits biological productivity^9,10^.

Atmospheric deposition of leachable metals can represent an important source of metals to the open ocean, in conjunction with other sources. Atmospheric aerosols are solids or liquids suspended in the atmosphere, and have diverse sources, sizes, and chemical composition^11^. Desert dust eroded from dry, unvegetated regions during strong wind events, is by mass one of the most important aerosols, and dominates as the source of many important trace metals. However, only a small fraction of metals in desert dust are soluble and available to the ocean biota^12,13^. For example, studies have suggested that although most of the total iron is contained in dust, much of the soluble (bioavailable) iron derives from combustion sources of iron^14,15^, even in the remote Southern Ocean^16^. There is also evidence that the iron in dust is processed by acids in the atmosphere to become more soluble^17^. Because humans are increasing combustion emissions of metals to the atmosphere^18^, contributing to atmospheric acidity, and potentially causing an increase in desert dust emissions due to land use and climate change, anthropogenic activities are likely to be increasing the deposition of soluble metals, especially iron, to the ocean^19^. In the HNLC areas, there is a direct link between atmospheric iron inputs and biological production and carbon export^20,21^ (Fig. 1a). Some phytoplankton (diazatrophs) are capable of nitrogen fixation^22^, and they are often growth-limited by the availability of iron or phosphorus in the oligotrophic ocean regions where the other phytoplankton are nitrogen-limited^23,24^ (Fig. 1a). Thus, deposition of soluble iron can impact rates of nitrogen fixation and community carbon export^23,24^, although at times, the impacts of iron deposition will be delayed^25^. Aerosol nutrient inputs can influence the biological export rates above the ocean oxygen minimum zones, thereby influencing rates of water column denitrification^26^. Thus, both nitrogen fixation and denitrification are modified by atmospheric nutrient inputs, and iron availability critically modifies the dynamic feedbacks between these key nitrogen cycle fluxes and the carbon cycle^27,28^ (Fig. 1a). Over longer timescales, the input of nutrients from the atmosphere strongly impacts the large-scale patterns of nutrient limitation and biological productivity in the oceans (Fig. 1b). Our understanding of aerosol metal leaching and the resulting impacts on ocean biota has experienced substantial improvements in recent years. This review addresses the important couplings between aerosol metals and ocean biology. First, the sources of metals to the atmosphere and atmospheric processing of these metals is presented. Then the short and long-term impacts of this on ocean biogeochemistry are discussed, as well as the best steps forward to improve our understanding of aerosol-biota interactions. Two boxes supplement this information with a discussion of new observational methods for aerosols and ocean metals, and another box focusing on the solubility of aerosols.

### Sources of aerosol metals

**Fig. 2:**
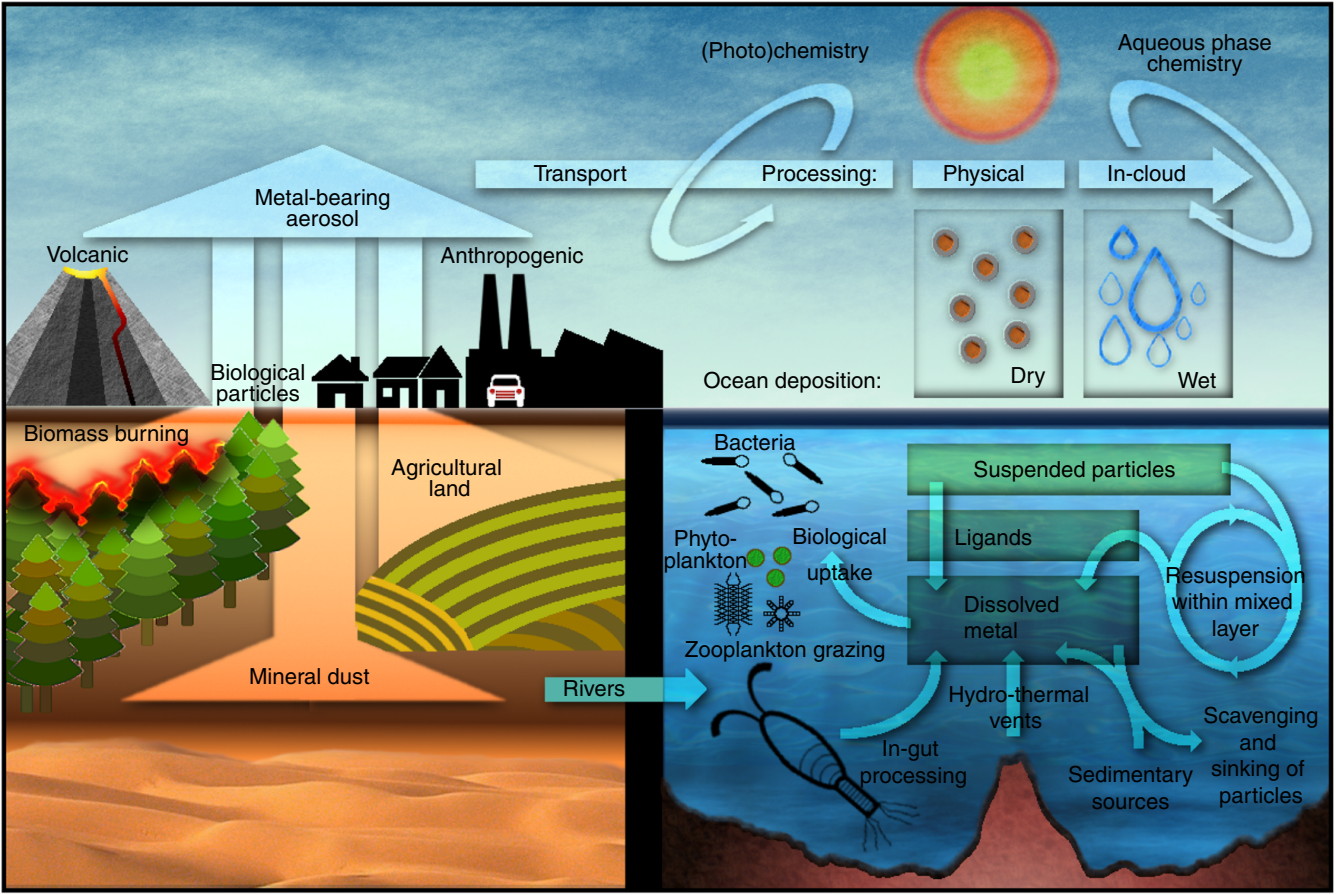
Schematic of aerosol metal dissolution and impacts on the ocean. Metal-bearing aerosols are emitted from natural (mineral dust, volcanoes, biological particles, and biomass burning) and anthropogenic activities (industrial combustion, agriculture, and land conversion), mostly over land. Combustion sources tend to have more soluble metals than the larger mineral dust sources. Some of the aerosols are transported to ocean regions, and can become processed so that the metals are more soluble by acids, photochemical and/or cloud processes, before being deposited to the oceans. In the oceans, the metals in the particles experience different chemical conditions, and can become more or less soluble, due to changes in acidity and ligands, for example. The metals can be cycled through bacteria, phytoplankton and zooplankton, and/or sink through the ocean to be scavenged in the deep ocean, or be deposited into the sediment. Sedimentary sources, riverine inputs, and hydrothermal vents can be important for many metals, as sources of new metals in the oceans

**Fig. 3:**
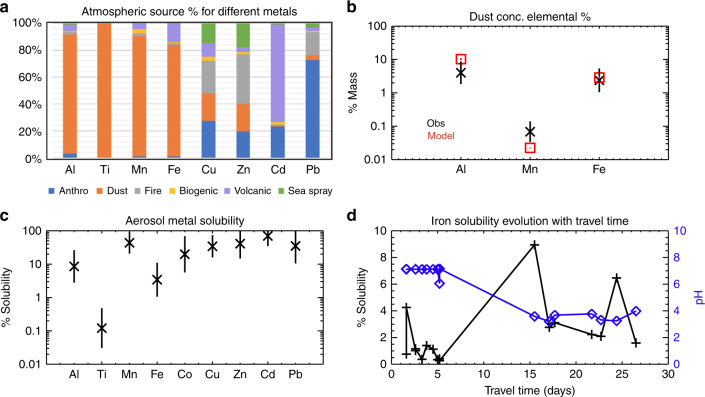
Variations in aerosol metal composition, solubility, and evolution. **a** Estimates of the percent of the atmospheric source for different metals; **b** estimates of the mass percentage of different metals in dust concentrations, from observations and model simulations in dust storm events (from ref. ^35^). **c** Measurements of metal solubility from ocean cruises with standard deviations shown as error bars^12,36^. **d** Measurements of iron solubility and pH (blue) as a function of travel time from the source region from ocean cruise (data taken from ref. ^44^)

**Fig. 4:**
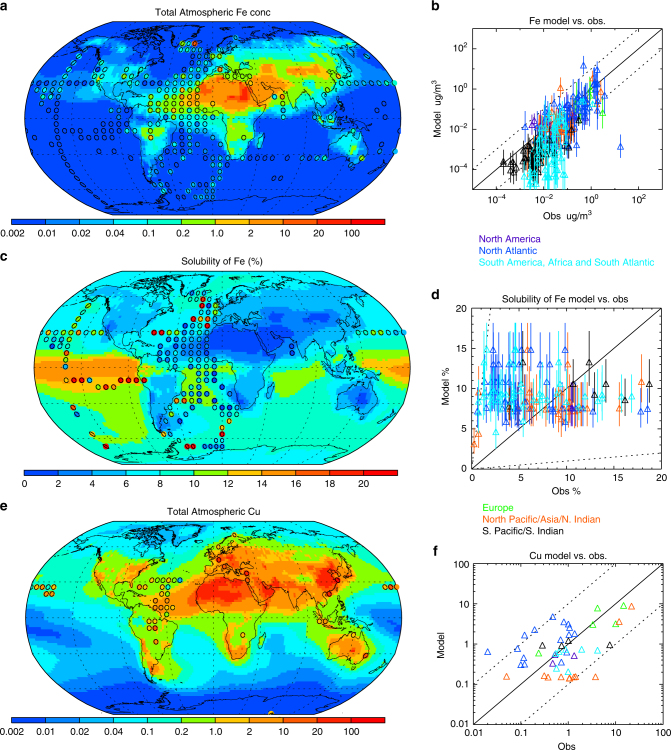
Spatial distribution of metals in aerosols. Distribution of iron concentrations (R. Scanza et al. (manuscript in preparation) and ref. ^160^) (**a**), iron solubility (R. Scanza et al. (manuscript in preparation) and ref. ^160^) (**c**), and copper concentrations^10^ (**e**) in models and observations. Iron solubility in measurements in the atmospheric aerosols (**a**) and modeled values (**b**), and scatter plot comparing the observations versus the model for total iron (**b**), solubility of iron (**d**), and copper (**f**). Vertical bars in **b**, **d** represent one standard deviation above and below the annual mean values, based on daily averages within a chemical transport model. The mean and standard deviation are calculated in log-normal space, as the distribution of the values is closer to a log-normal distribution than a Gaussian distribution^161^

Available estimates suggest that the aerosol sources of metals are dominated by desert dust (mineral aerosols) for Al, Ti, Mn, and Fe, but combustion sources may also contribute to those elements and may be especially important for Cu, Zn, Pb, while Cd sources may be dominated by volcanoes (Fig. 2; Fig. 3a; Table 1). There are limited studies looking at the atmospheric cycle of metals besides iron, and, except for iron and copper, there has not been a comparison of model predictions of metal distributions with observations (Fig. 4a, c), and so these aerosol source budgets must be considered uncertain (Table 1; Fig. 3a). Once airborne, coagulation of aerosol particles and the condensation of low volatility gases onto particle surfaces results in a continuing evolution of the particles’ composition^29,30^. Deposition to the ocean can occur via direct (dry) deposition through gravitational or turbulent mixing of the particle, or through rain-out (wet deposition) after the particle has become entrained within a precipitating cloud droplet (Fig. 2). Comparisons with available observations suggest that even for metals like Cu, with substantial anthropogenic sources (Fig. 3a), ocean regions downwind from the desert dust sources of North Africa will experience high atmospheric inputs (Fig. 4a, e). Volcanoes can be important sources during explosive events^31^, and for Cd generally, but quiescent volcanoes generally represent only locally important sources for metals (Table 1).

**Table 1:** Aerosol metal sources to the atmosphere

	Anthropogenic	Dust	Fire	Biogenic	Sea spray	Volcanic	Total
Al	3000	80,000	2000	200	1000	5000	90,000
Ti	2	8000	6				8000
Mn	10	900	20	30	2	40	1000
Fe	700	50,000	1000	200	200	9000	60,000
Cu	30	20	20	3	10	9	100
Zn	60	60	100	5	50	10	300
Cd	3	0	0	0.2	0	9	10
Pb	100	6	30	2	5	4	200
Sum	4000	14,000	3000	400	1000	14,000	160,000

Sources are reported in Gg/year^18,127^. For this table, the calculations are done using as many digits as possible, and then the values are presented using one significant digit to illustrate the lack of confidence in the values. The same values are used for Fig. 3a

The magnitude of dust emissions is related to vegetation cover, the soil characteristics (including particle size, composition, and moisture content) and prevailing meteorological conditions^32^. Subsequent long range transport of dust to the open ocean is well documented in marine observations, and some of the earliest observations were made by Charles Darwin over a century ago^33^. Dust emission, transport, and deposition events are highly episodic in nature, with 30–90% of annual average dust deposition occurring over just 5% of the days of a year^32^. The amount of metals in crustal material and soils is highly variable by element type (Fig. 3b), and for different elements can vary by region due to the different geological influences^34^, and thus vary in desert dust from different regions^35^ (Supplementary Figure 1). For example, there is more Al than Fe in most dust regions (Fig. 3b). The solubility of aerosol metals is also important when considering the potential biological impacts of deposition (see Boxes 1 and 2 for more discussion of solubility). Observations over ocean regions suggest quite different solubilities for different metals, with iron tending to be less soluble than many other metals, such as Mn or Co, but more soluble than Ti^36,37^ (Box 1; Fig. 3c). The variability in metal solubility is likely to be driven by both variability in sources and atmospheric processing (Box 2). Indeed, there is evidence that combustion sources of aerosols may be much more soluble than dust sources, so although their total elemental emissions may be small, combustion emissions of metals may supply more bioavailable metals than dust, especially in regions with high emissions (Tables 1 and 2; Box 2).

**Table 2:** Soluble metal emissions

	Iron emissions /Tg a^-1^	Soluble iron emission /Tg a^-1^ (solubility)	Soluble iron source /Tg a^−1^	Observed iron emission solubility
Dust	57	0.51 (1%)	0.54	<1–4%^59^
Fire	1.2	0.048 (4%)	0.066	0.5–46%^22,68^
Industry	0.66	0.026 (4%)	0.054	0.2–25%^65,66^
Shipping	Additional 2%	—	—	36–81%^61,64,65^
Atmospheric iron	—	—		0.01–80%^19^

Rates are reported in Gg/year (R. Scanza et al. (manuscript in preparation) and ref. ^38^)

The most studied metal in aerosols is iron, as it is known to be a limiting micro-nutrient. The largest concentrations of iron are observed downwind of the major desert regions (Fig. 3a), similar to Cu (Fig. 4e). However, only a fraction (0.1–90%) of the iron in aerosols is soluble when deposited to the open ocean (Fig. 3c; Box 2), with relatively low solubilities observed close to the desert dust sources, and higher solubilities in remote or polluted regions when iron concentrations are low, although there is substantial temporal and spatial variability (Fig. 4c; Box 2). This variability of iron solubility is likely to be due to three main factors: the aerosol source, atmospheric processing, and the characteristics of the receiving sea water (Fig. 2; Box 2).

Anthropogenic fossil fuel combustion and biomass burning activity has created a new source of combustion Fe to the atmosphere, while wildfires present a natural source of combustion Fe^14^ (Table 1). Although emissions of combustion Fe are 1–2 orders of magnitude smaller than mineral dust Fe (Table 1), they could account for up to 50% of the total soluble Fe deposited in remote HNLC ocean regions^15^ (Fig. 1c) and be important globally (Table 2). The spatial distribution of Fe emissions from fossil fuel combustion, anthropogenic biomass burning, and wildfires contrasts with that of dust^15^ reflecting underlying patterns in human population density, shipping, and vegetation distributions. Shipping contributes a relatively small (~2%) source of Fe^38^; however, the solubility of oil combustion-derived Fe is significantly higher (36–81%) compared to coal (0.2–25%)^39^. Shipping-derived Fe is largely deposited in the nearby oceans and is therefore a significant source (up to 50%) of bioavailable soluble Fe for remote northern hemisphere and equatorial Pacific HNLC ocean regions^40^. Observations of Fe from wildfires are limited and emissions are currently based on estimated Fe:black carbon ratios^14,41^. Modeling suggests that over the southern hemisphere fires represent a significant source of soluble Fe, which could be equal to that from mineral dust (Fig. 1c). Present day Fe emissions from fires are estimated to be approximately double those of industrial combustion Fe emissions^24^, but could increase to an order of magnitude larger in the future as fire occurrence increases and industrial emission decrease^42^. Observed solubilities of Fe from fires range from 0.5-46% ^14,43^, reflecting a significant source of uncertainty in their impacts on soluble Fe deposition.

### Box 1 Observational methods and GEOTRACES

Recent observational studies have transformed our understanding of aerosol metal leaching and their impact on ocean biota, and here we highlight the innovations in these observations, using the example of GEOTRACES. GEOTRACES is an international field campaign designed to “identify processes and quantify fluxes that control the distributions of key trace elements and isotopes in the ocean, and to establish the sensitivity of these distributions to changing environmental conditions” (http://www.geotraces.org/). GEOTRACES extensive intercalibration efforts have led to standardized techniques to minimize contamination and ensure that multiple laboratories achieve the same accurate results, regardless of method^139^. GEOTRACES is also providing large-scale measurements of iron-binding ligands^69,140^, the trace element content and stoichiometry in different plankton groups^141^, novel methods to assess the depositional flux of metals to the ocean^142–144^. An important complement to the large-scale surveys from GEOTRACES are timeseries measurements from a single location, which can help constrain trace metal sources and sinks^145,146^ and process studies to help constrain trace metal inputs and the biotic and abiotic ocean processes^5^. We focus here on a few of the advances in observations.

Leaching experiments measure fractional solubility by comparing the concentration of a metal in a soluble extract to the total in the bulk aerosol material. Total metal content is often determined by digesting the aerosol sample under heated conditions with hydrofluoric and other acids, followed by quantitation on an inductively coupled plasma mass spectrometer^147,148^. Metal solubility measurements are made by extracting in pure water, sea water, or a buffered acidic solution that replicates rain water. The chemical characteristics of these solvents, principally pH^149^, affects metal solubility. In the case of using natural sea water to perform extractions, metal solubility is additionally affected by background concentrations of organics^3,150^, dissolved trace metals^151^, superoxide^152^, and hydrogen peroxide^96^ that may be naturally present in the sea water and vary depending on the sea water source. However, for Fe, there appears to be greater variability in solubility between aerosols from different sources, than between different leaching solutions^153^. Extraction time likewise varies from on the order of seconds, as when filter aerosol samples are extracted by a solvent using a filter funnel under vacuum^154^, to longer timescales (minutes to hours) that have been used for simple batch leaching methods^147^, sonicated samples^147^, and semi-continuous flow-through approaches^153^. Comparison of prolonged leaching over several days to instantaneous leaching shows different dissolution behaviors for different metals^155^, with Zn, Co, and Cd dissolved rapidly in sea water and Ni, Al, Cu, and Mn dissolved gradually over time.

The effect of aerosol nutrients on biota can be measured experimentally via bottle incubation experiments and mesocosm studies. Bottle incubations measure the responses of discrete populations at a precise location over a period of hours to days. In these studies, natural microbial communities in sea water are collected and dispensed into bottles, and aerosol additions are made by adding particles (on filters or not) or leachate. Following additions, the bottles are incubated, typically under ambient conditions, and the community response is monitored over time. Mesocosm experiments are similar to bottle incubation experiments, but generally use a larger volume of sea water incubated in situ for a longer period of time^95,96^.

Large collections of GEOTRACES datasets and an electronic digital atlas for visualization were released in the GEOTRACES Intermediate Data Product 2014 (IDP2014) and were updated in the Intermediate Data Product 2017^156^.

### Box 2 Understanding the relationship between total Fe and solubility of Fe in aerosols

Observational studies have suggested an inverse relationship between total iron mass concentrations and the solubility of the iron^12^. This relationship is often used as a constraint on model simulations, to better understand the sources and processes relevant for atmospheric soluble iron^38,157^. However, here we show why the inverse relationship could be due to either different types of sources, or due to atmospheric processing, using a simple 1-dimensional plume model. We remove the extra uncertainty that the mixing of different air masses introduces on solubility during transport in order to assess the relevant processes within a single plume environment.

For the first case (combustion), we assume that both dust and combustion sources of iron are present, but that the initial emissions of combustion iron contain some soluble iron, but dust iron is insoluble. We assume that dust contains most of the mass of iron emitted to the atmosphere, and due to gravitational settling of the relatively larger dust particles, it has a shorter atmospheric lifetime than the smaller combustion aerosols. These assumptions are consistent with current understanding of these two aerosol types^11,15,38^. We also assume that the combustion and dust sources come only from land regions, and during transport undergo dispersion and deposition, thus reducing in concentration with time (a). The longer lifetime of the smaller, more soluble, combustion aerosols results in a slower decay in concentrations with respect to time, allowing the solubility to increase with the added residence time compared to dust (panel a, blue case). For a second case (simple atmospheric processing), we assume that the iron is released at a low rate of solubility at source, but is processed in the atmosphere with time, becoming gradually more soluble (panel a, green case). For our third and final case, we assume that the atmospheric processing occurs due to a pollutant (e.g., sulfur-based or organic acid) emitted on land as well (gray line), which decreases in concentration with time. This suggests a similar increase in solubility with time as in the previous cases (pollutant-based atmospheric processing case: red line). Panel b shows that any one of these idealized cases can provide a solution, which is within the large range of the observations. Since combustion iron is much more soluble, but less abundant, the solubility of iron in aerosols close to source regions is smaller, but increases quickly. Solubility increases less quickly away from source regions if the pollutant is required for atmospheric processing, because it is decreasing away from source regions. The exact slope of these lines is sensitive to the assumptions made, but each of these cases could match available observations within the broad range measured. Thus, knowing that there is an inverse relationship between total iron and percentage solubility does not constrain which of these cases is more likely to represent the actual metal dissolution processes that the aerosol undergoes during transport^49^.

In addition, this simple case explains why there is not an observed correlation between measured concentrations of acid compounds in pollution and solubility even if the pollutant is responsible^158^. There is an anti-correlation between solubility and pollutant concentrations close to source, although both evolve slowly far from the sources (red-dashed line versus the gray line). This is because not only the presence of a pollutant is required to process the iron, but also time, and thus the pollutant decays with time, even as it adds to the solubility. This is consistent with detailed model simulations, which include mixing of different air masses and suggest there is no correlation between the pollutant concentration and solubility of the iron at remote locations^84^.



Another difficulty in interpreting the observations is that they are often taken onboard ship cruises, and thus only represent a 1–2 day average snapshot of the atmospheric aerosol concentrations. Unfortunately, observations over remote regions on longer timescales are very limited due to the complex logistics required in collecting them^16,159^. Because of the strong variability in both the total iron and its solubility, the chemical transport model predictions suggest almost an order of magnitude variation in total iron concentrations, and a smaller, but still significant amount of variability in the % soluble (c). Thus, more observations, at fixed stations^16,159^ or with repeat transects analyzed to create climatologies^36,160^, are required to provide more insight and constraints on the characteristics of aerosol metal dissolution in remote ocean regions.

### Atmospheric processing and deposition of metals

Aerosol metal solubility is influenced by atmospheric processes that occur before aerosol particles are deposited on the ocean surface. These atmospheric processes can be quite variable across different metals; however, most studies have focused on iron. In remote regions, the solubility of iron can be quite high (Fig. 3b), and observations suggest that under some circumstances iron solubility in dust increases as it travels downwind^44^ (Fig. 3d). Several chemical mechanisms have been proposed to explain the increase in solubility. Many studies have shown that increases in acidity (decreases in pH) will result in more iron being solubilized^17^. Studies have highlighted the role that anthropogenic pollution can play in solubilizing iron^17,19^. Aerosol water can become very acidic because of the low volatility of sulfate and organic acids and thus may promote some chemical reactions converting metals to a more soluble state^44^. The presence of organic compounds, such as oxalate, in cloud water can promote photo-reduction^45^ and could increase iron solubility by up to an additional 75% compared to just acidic dissolution^46^. On the other hand, dust can partially buffer acids, for example, if calcium carbonate is present within the particle^47^. Some observations suggest that wet-deposited iron tends to be more soluble^13^, and may lead to increased productivity once deposited in the oceans^48^. This may be because of in-cloud acid processing^17^ or because organic ligands may stabilize soluble metals once dissolved in cloud drops^45^. The chemical composition of aerosols likewise changes as the size distribution shifts toward smaller particles, with larger, less soluble dust aerosols giving way to smaller, more soluble combustion aerosols (see Box 2 for more discussion).

**Fig. 5:**
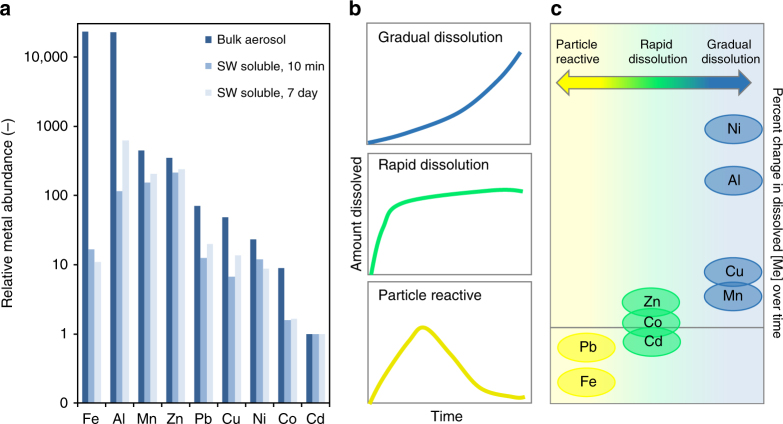
Ocean dissolution processes. **a** Elemental molar fractions of metals in bulk aerosol material collected In Eilat, Israel along the coast of the Gulf of Aqaba, Red Sea, and in the sea water-soluble fractions after 10 min and 7 days of leaching. Metals are arranged from most to least abundant based on the abundance in the bulk aerosol. Data were published previously in Mackey et al.^74^; see reference for sample collection and methodological details. **b** Three dissolution modes were observed for different metals. Gradually dissolving metals show increased dissolved concentrations over time, whereas nearly all soluble metal is released immediately for rapidly dissolving metals. For particle reactive metals, the dissolved concentrations first increase but then decrease due to sorption onto particles and/or wall loss. **c** Schematic of the percent change in soluble concentration of several aerosol metals over time based on measurements after 10 min and 7 days of leaching. Positive values (above the horizontal line) indicate more metal dissolves with longer leaching (gradual dissolution), while negative values (below the horizontal line) indicate declining concentrations with time (particle reactive). Values near zero indicate rapid dissolution where the concentration of dissolved metal remains relatively constant over time

The complexity of explaining observed metal solubility in aerosol concentrations and deposition can be illustrated by the better studied Fe example, although metal chemical behavior can be hetereogeneous^49,50^. No single process has been identified as the dominant controlling factor for Fe aerosol solubility, and it is likely that combinations of competing factors are important depending on the region and season. The observed inverse relationship between amount and solubility of iron (and other metals)^12^ can be explained through any of the above mechanisms (Box 2). From the spatial distribution, one can see that the lowest solubilities occur close to the dust source regions (Fig. 5c), but there is substantial variability in the observed solubility even at nearby locations. Models can simulate much of the spatial variability in solubility, but cannot reproduce the very high solubilities sometimes observed. (Fig. 4c, d). Model results suggest that single daily averaged observations are difficult to use to constrain the solubility, since there is considerable temporal variability in the solubility and the amount of iron at a specific location (Fig. 4c; Box 2) (R. Scanza et al. (manuscript in preparation) and ref. ^38,42^). While acid processing is hypothesized to increase soluble iron, there is rarely correlation in the observations between acid concentrations (e.g., sulfate) and iron solubility; however, modeling studies have shown that even if the only mechanism for the production of soluble iron is sulfate processing, because of the need for time to solubilize the iron, there is no correlation^15^ (Box 2), suggesting that this lack of correlation is not helpful in constraining the source of soluble iron in the atmosphere. Model calculations suggest that for most ocean basins, the upwind dust source is the dominant source of soluble iron (Fig. 1c). It is important to note that the dominant sources of total iron are different than the dominant sources of soluble iron, and that in the Southern Hemisphere, combustion sources may dominate over dust sources for soluble iron (Fig. 1c; Box 2). A new potentially transformational method of determining the source of iron or other metals is isotopic analysis, which could discriminate between combustion and dust sources^52^, in addition to using spatial variability in models and observations (Fig. 4d).

Global synthesis of estimated sources, modeled distributions, and comparison to observations, which would allow for a rigorous assessment of the atmospheric budget have only occurred for Fe and Cu^53^ (Tables 1 and 2; Fig. 4). However, the high quality of the available data (Box 1), with interesting spatial patterns^35^ and potentially large anthropogenic sources (Table 1), suggest more studies should occur.

### Short-term impacts of aerosol metal deposition to the ocean

Once aerosols are deposited on the ocean surface, dissolution of the metals is influenced by the physical, chemical, and biological characteristics of the receiving body of water. Metal dissolution can be either instantaneous or gradual, with Zn, Co, and Cd dissolving faster than other metals, and Ni, Al, Cu, and Mn dissolving slower^54^ (Fig. 5, Box 1). Fe and Pb tend to react strongly in water, showing an increase and then loss of dissolved metal (Fig. 5). Mixing and resuspension of particles within the water column increases their residence times, hence the particles have more time to interact with the water and for metals to dissolve, with smaller particles residing longer. The pre-existing concentration of the metal organic matter within the water also influences the rate of dissolution of additional metal from the particle^55,56^. For example, the majority of dissolved iron in the oceans is bound to iron-binding ligands or bound to colloids^57,58^. Sidephores are one ligand type produced by bacteria and are classified as strong binding ligands due to their high-conditional stability constants^59,60^. Weaker binding ligands in the upper water column have sources from remineralization of sinking organic matter^25^, the rupture of phytoplankton cells by viral lysis^61,62^, and zooplankton grazing^63^. Deeper water column ligand sources are thought to include refractory, humic-like substances^25,64–66^. The relative importance of these sources and sinks of metal-binding organic matter are unknown. As the understanding of the importance of trace metals other than Fe for biota has been documented in recent years, the role of ligands in controlling their distributions, inventories, and speciation in the ocean has gained recognition. Metals like cobalt^67,68^ and copper^69,70^ are examples of biologically important trace metals with speciation controlled by biogenic ligands. In the case of copper, ligand production may serve the dual purpose of maintaining adequate levels of free Cu for nutrition, while simultaneously preventing free Cu concentrations from reaching toxic levels.

In addition to ligand production, biota can influence metal dissolution directly via the processing of particles in their (acidic) vacuoles and guts, and by repackaging particles into fecal pellets, which affects the sinking rate and hence the amount of time the particle stays in the mixed layer^71^. Uptake of trace metals during growth can likewise have a strong effect on dissolved trace metal concentrations, which can become depleted in the water as they are taken up by cells. For example, phytoplankton have been shown to take up aerosol-derived trace metals like Fe, Co, Ni, and Mn rapidly over a period of days, drawing down their concentrations in sea water^70^. The extent to which biological uptake affects a given metal depends on the cellular quota for that metal, which varies among species and strains, and which can vary considerably within a species depending on availability of the metal^72^. For example, some cells have the ability to perform luxury uptake and storage of metals during periods of high availability. This has been observed for Fe, which *Trichodesmium*^73^ and *Synechococcus*^74^ both possess mechanisms to store within their cells for later use. Other removal processes from the ocean mixed layer include the scavenging (adsorption) onto sinking particles^75^. Both biological and physical removal processes scale positively with ocean productivity (most particles are biogenic) with rapid removal in productive regions.

**Fig. 6:**
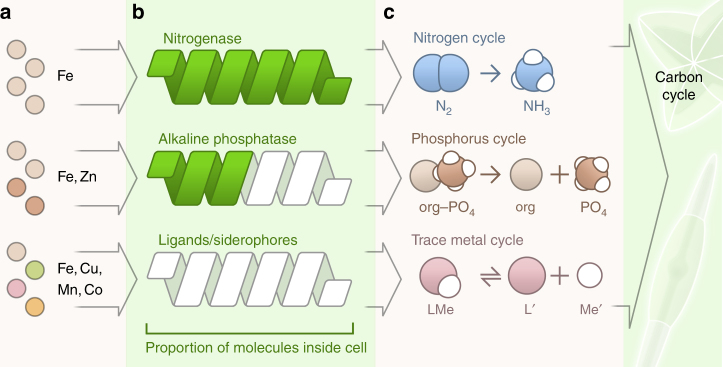
Biological responses of phytoplankton and bacteria to aerosol trace metals. Trace metals are required as co-factors for many biogeochemically important molecules. Certain aerosol trace metals (**a**) have been shown to stimulate production of nitrogenase^73,77^, alkaline phosphatase^91^ and metal-binding ligands^70,95,162^, and these molecules may remain within the cells (proportion of helix which is green) or be exuded into the sea water (**b**), where they catalyze chemical reactions that influence biogeochemical cycles (**c**). The cycling of N, P, and trace metals in turn affects the carbon cycle by influencing cellular growth rates. See Supplementary table 2 for compilation of studies, which this figure syntheses

Bottle incubation experiments were critical in first demonstrating that iron was the growth-limiting nutrient over the vast HNLC regions, which was verified by the subsequent larger-scale, in situ iron fertilization experiments^3,4^. Often, aerosol additions stimulate one or more populations by providing growth-limiting nutrients (Fig. 6; Box 1). This occurs because populations can be limited by different nutrients depending on cellular quotas and physiological requirements. Bottle incubation experiments have been used to identify differences in nutrient (co)limitation between communities in coastal versus offshore waters^70,76^, along open ocean transects^77,78^, over different seasons^79^, and among sites with varying amounts of anthropogenic influence^80^. For example, aerosol-derived Co, Mn, and Ni supported growth of oceanic (but not coastal) *Synechococcus* in the Sargasso Sea. Moreover, in both coastal and oceanic populations, moderate aerosol copper additions did not cause toxicity, and the production of Cu-binding ligands following aerosol Cu additions could indicate a nutritive role for Cu in the region. Most phytoplankton taxa appear capable of benefitting from aerosol-derived nutrients depending on their location and nutrient status. Indeed, diatoms, dinoflagellates, prymnesiophytes, picoplankton, diazotrophs, and heterotrophic bacteria have all been shown to respond favorably to aerosols in different locations (Supplementary Table 2). Size-fractionated phytoplankton biomass in the Western Philippine Sea had high concentrations of several trace metals (Fe, Mn, Zn, and Cu), which was attributed to the high anthropogenic aerosol deposition in the region^81^.

Diazotroph responses to aerosol additions are well studied due to the essential role of these cells in the marine nitrogen cycle as providers of new bioavailable nitrogen. Most studies focus on the effects of aerosol Fe, because the nitrogenase enzyme that catalyzes nitrogen fixation has a high Fe requirement. Many genera have been observed to benefit from aerosol additions, including *Trichodesmium* spp.^73,82^ and unicellular cyanobacteria^77^, although the response is geographically variable. For example, in the ultraoligotrophic Southeast Pacific Ocean, N_2_ fixation was not enhanced by aerosol additions due to the very low abundance of diazotrophs at the site^78^, and in many locations of the Mediterranean Sea, N_2_ fixation is spurred by aerosol P rather than aerosol Fe^83,84^. These studies have also contributed to our understanding of how phytoplankton access Fe in the environment. *Trichodesmium* performs luxury uptake of Fe that is in excess of its cellular requirement following aerosol additions^7^, and has been shown to trap and move dust particles within its trichomes to accelerate dissolution and facilitate uptake^82^.

Recent studies have begun to focus on the responses of heterotrophic bacteria following aerosol additions. As with phytoplankton, changes in heterotroph abundances and bacterial production rates following aerosol additions are site specific. In the majority of cases, the heterotrophic response to aerosols are stronger^85–89^ than the autotrophic response, although one study reported a weaker response^90^. Aside from measuring microorganisms themselves, some bottle incubation studies quantify important biogenic enzymes or molecules that microbes synthesize in response to aerosol additions (Fig. 6). In many cases, these products influence major biogeochemical cycles. Nitrogenase is one such example, as it plays an essential role in the nitrogen cycle, since it catalyzes nitrogen fixation. The phosphorus cycle is likewise sensitive to aerosol metals because the alkaline phosphatase enzymes responsible for cleaving phosphate groups from organic molecules utilize iron or zinc as co-factors. Addition of iron and zinc from aerosols has been shown to stimulate alkaline phosphatase activity in the North Atlantic Ocean^91^, although the effect is less pronounced in the eastern Atlantic, presumably due to the considerably higher amount of Saharan dust and associated metals already present in the water^92^. Similarly, alkaline phosphatase activity is not enhanced in the Gulf of Aqaba, Red Sea, which receives high levels of dust deposition^93^. Finally, metal-binding ligand production can be measured in bottle incubation experiments to assess the role of biota in modulating dissolved metal concentrations following aerosol enrichment (Fig. 6). For example, Cu- binding ligand concentrations were observed to change over a period of days during an incubation in the Sargasso Sea^94^, and increased iron dissolution over time in a Mediterranean Sea mesocosm study was attributed to ligand production during phytoplankton growth^95,96^ (though Fe-binding ligands were not directly measured in those studies). Studies that quantify ligand concentrations and stability constants during incubations are still rare, possibly due to the highly specialized methodology required, but necessary for future progress in understanding the trace metal cycling.

In addition to increasing our understanding of the fertilizing effects of aerosols on marine microbes and their biogeochemical activity, bottle incubation experiments have also been instrumental in demonstrating the potential toxic and interactive effects that aerosol metals may have on phytoplankton growth and community composition. For example, Mann et al.^97^ proposed that the differential copper toxicity thresholds and depth distributions among *Synechococcus* and *Prochlorococcus* ecotypes in the North Atlantic Ocean was driven by the higher dust-derived Cu concentrations in surface waters. The role of aerosol Cu as a toxicant was later empirically confirmed through bottle incubation experiments in the Red Sea, where picoeukaryotes and *Synechococcus* populations declined when aerosol Cu exceeded their toxicity threshold^10^, and in the South China Sea, where *Prochlorococcus* and *Synechococcus* populations declined following the strongest deposition event^80^. Later studies used a combination of bottle incubation experiments, remote sensing, and direct aerosol sampling to show that phytoplankton copper toxicity from aerosols in the East China Sea was mitigated when aerosols contained a higher proportion of iron^98^. Both the toxicity of copper and its mitigation by other metals like iron and manganese had been well studied using laboratory cultures, but the bottle incubation experiments demonstrated their role in natural populations. Mesoscale enclosures (mesocosms) of ambient sea water allow for manipulation of environmental conditions and nutrient inputs, and the study of larger-scale processes than bottle incubations, including more realistic food web dynamics and interaction with grazers and the export of sinking material^95,99^. The effect of iron from Saharan dust has been assessed via direct measurement in the high deposition eastern North Atlantic Ocean^100^, as well as in the more moderate deposition western North Atlantic^101^.

Remote sensing of aerosol optical thickness has been used to explore the longer term impacts of atmospheric deposition on phytoplankton in the Mediterranean Sea^9^, coastal California^102^, and Chinese marginal Seas^103^. However, an important caveat is that satellite ocean color errors are correlated with atmospheric aerosols^104^, and aerosols observed in the atmosphere are not the same aerosols that are deposited in the ocean^105^. In addition, it is difficult to tease apart whether the observed response results from N, iron, or other micronutrients in the aerosol. Because of these difficulties, as well as the longer timescales of the effects of atmospheric deposition, it may be difficult to detect and attribute responses to trace metal deposition in remotely sensed data^25^.

### Long-term biogeochemical impacts of aerosol metal deposition

The large-scale distributions of other key trace metals with atmospheric sources are providing insights into the magnitude of atmospheric sources, the roles of trace metals in biogeochemistry, and the potential for limitation or co-limitation of phytoplankton growth by cobalt^80,106,107^, manganese^6,80,108^, zinc^107,109,110^, and potentially other trace metals^111^ (Box 1; Fig. 6). Strong biological drawdown of zinc in Southern Ocean surface waters can reduce the northward flux into the thermocline of the South Atlantic gyre, which likely selects for plankton with lower Zn requirements^110^. Contrasting observations from the North and South Atlantic basins strongly suggests that biological uptake drives the low surface values in the North Atlantic, and that Zn and/or Co availability can limit the ability of plankton to utilize organic phosphorus via alkaline phosphatase enzymes, which require these metal co-factors^106,107^. Volcanic ash was suggested to enhance productivity more than iron-only additions due to relief of manganese co-limitation^6^. The growing global observational database for manganese has facilitated the development of initial model simulations of the global marine manganese cycle^108^.

Efforts to include the influence of atmospheric trace metal deposition into marine ecosystem and biogeochemical models remain largely focused on iron deposition (Table 1). These efforts are hampered by large uncertainties in the magnitude of dissolved iron inputs from different sources, and a still-limited understanding of the interactions with the iron-binding ligands that dictate the losses to particle scavenging (Fig. 2; Table 3). A recent inter-comparison of global-scale, marine iron cycle models revealed a great disparity in the magnitude of iron inputs to the ocean from different sources across the models^112^ (Table 3). Atmospheric deposition of soluble iron to the oceans ranged from 1.4 to 32.7 Gmol/year, with even larger variability in the assumed inputs from sedimentary sources. Atmospheric deposition and marine sediments have been recognized as important sources of dissolved iron, but there is still great uncertainty as to their relative contributions to the open ocean. Some evidence suggests atmospheric deposition is an important contributor to metal concentrations in the regions of the ocean where the biological demands are greatest^21,113,114^. Recent observations from the GEOTRACES program illustrate the importance of hydrothermal vent systems as a major deep ocean source for iron and other trace elements^115^. In addition to these three major dissolved Fe sources, inputs from river, glacier, iceberg, sea ice, and volcanic ash sources can be important regionally^31,116,117^, although they are not important globally (Table 3). Mn ocean sources are estimated to be dominated by hydrothermal vents^108^ (Table 3). While reviews are available across multiple metals^5^, budgets for other metals are not available (Table 3). Nutrient concentrations may increase notably in response to a single deposition event, which can then influence species composition and productivity, both for phytoplankton and heterotrophic bacteria^118,119^. While large changes in deposition are likely on daily to decadal scales, models used in the Fifth Phase of the Coupled Model Intercomparison Project (CMIP5) included only monthly averaged, climatological Fe deposition, which suggests they underestimated the potential biogeochemical impacts of changing deposition with climate change^120^.

**Table 3:** Metal sources to the ocean

	Deposition	Sediment	Hydrothermal	Riverine	Sum
Fe	700	4000	800	80	5000
Mn	300	200	6000	20	6000

Sources are reported in Gg/year. Only values for Fe and Mn are available^21,108^

There is variability in phytoplankton trace metal quotas for different trace metals^77,121^. Thus, the stoichiometry of the trace metals and macronutrients in atmospheric deposition may modify community composition by differentially supporting different species^80,121^. For example, in the Caribbean Sea near Barbados, an area of intense deposition, nitrogen and iron levels are consistently elevated relative to phosphorus. This selects for a population dominated by *Prochlorococcus*, which has lower phosphorus requirements relative to other phytoplankton, and that is efficient at taking up the ammonium released from the aerosols^122^. In contrast, in the Equatorial Pacific HNLC, low-dust deposition coupled with relatively low dissolved iron concentrations in upwelled water create an intense iron deficit that favors microbial populations capable of performing vigorous iron recycling to fuel the high observed nitrate consumption rates^123^.

The availability of trace metals over evolutionary timescales may place a selective pressure on microorganisms to evolve novel adaptations for acquiring, utilizing, and/or avoiding toxicity from trace metals, and these effects can manifest in individual species or strains, as well as at the community and ecosystem levels. In the North Atlantic Ocean, the pattern of Saharan dust deposition establishes a gradient with consistently high iron concentrations in the east and lower latitudes, and more variable iron concentrations in the west. This gradient in iron availability has been linked to the retention and expression of iron acquisition, storage, and regulatory proteins in *Synechococcus*, where a strain from the variable western region has more genetic flexibility to respond to changes in iron availability^74^. A related strain from the consistently high deposition region lacks this flexibility, likely because it imparts no selective advantage given the relatively consistent iron supply, and because retaining these proteins/genes requires nitrogen and phosphorus in an environment where these elements scarce and often co-limiting. Similarly, *Prochlorococcus* from the iron-limited Equatorial Pacific HNLC region minimizes iron requirements through genomic streamlining in which iron-containing metalloenzymes are eliminated from the genome^124^. Oceanic diatoms from iron-limited regions receiving low-dust deposition have likewise adapted genetically by utilizing copper rather than iron for certain essential proteins^125^, and alkaline phosphatase activity in the North Atlantic Ocean has been linked to aerosol iron and zinc deposition^91,92^, as these metals are co-factors in the alkaline phosphatase enzyme.

### Aerosol metal deposition and leaching under future climates

Because of increases in anthropogenic emissions of combustion iron and acids, modeling suggests that soluble iron deposited to the oceans has increased by a factor of 2–3 since the pre-industrial era^19,38,42^. Despite clean air acts reducing anthropogenic gas emissions, calculated pH levels from observed continental aerosols has remained constant at between 0 and 2 over the last 15 years^126^, suggesting that acid dissolution processes will remain an import process in controlling the availability of soluble Fe to the oceans into the future, even with stricter air pollution regulations. In addition, desert dust may have almost doubled over the 20th century, due to land use and/or climate change^19^. Thus, large changes in soluble iron inputs to the ocean are likely to have occurred. Future soluble iron deposition is uncertain depending on the relative importance of dust, combustion iron, and acidity, and their potential changes^38,42^. No projections are currently available for other metals, but estimates of the anthropogenic component of the atmospheric sources (Table 1), suggest that many metals may be heavily modified by humans^18,127^, and large changes may occur in the future. Ocean acidification has the potential to alter metal sea water chemistry, resulting in changes to the dissolution rate of metals within the ocean column^128^.

Despite these sources of uncertainty, several trends are expected to emerge as the surface ocean warms in response to climate change. Increased thermal stratification, particularly at mid and high latitudes, will cause the mixed layer depth to shoal, which could increase the concentrations of aerosol-derived metals in surface waters. However, this process would be less pronounced at low latitudes where aerosol (from dust) flux is highest, due to a more modest shoaling of the mixed layer in the tropics. All of the CMIP5 ocean biogeochemical models projected consistent patterns of increasing sea surface temperature and stratification, and declining ocean productivity over the 21st century under strong warming scenarios^129^. As the upper ocean warms and stratifies, the upward nutrient flux declines, and global-scale net primary production and the biological export of carbon to the ocean interior consistently decreased across the models^129,130^. The reduced nutrient flux from below will increase the relative importance atmospheric nutrient inputs to the oceans.

The effect of toxicity from the potential increase in surface metal concentrations has not been explored in models because iron is currently the only trace metal that is routinely included in model simulations, and iron does not exceed toxicity thresholds in sea water. However, the projected range expansion of small phytoplankton like *Prochlorococcus* and *Synechococcus*, which have the highest sensitivity to trace metals like copper^10,131^, suggests that the increase in surface water metal concentrations could lead to metal toxicity in more areas of the ocean in the future. This effect would be heightened if anthropogenic aerosol emissions increase, due to their higher trace metal content and solubility.

### Future perspectives

In this review, we have synthesized available observations of aerosol metals deposited in ocean (Fig. 2), their potential to be leached in oceans (Fig. 5), their short-term impacts on ocean biota (Fig. 6), and their long-term impacts on ocean biogeochemistry (Fig. 1a, b; Box 1). There is substantial literature on many important metals including Al, Ti, Mn, Fe, Co, Cu, Zn, Cd, and Pb. Many of these metals have seen a substantial increase in their atmospheric sources due to anthropogenic activities (Fig. 3a; Table 1a), but only Fe has been extensively studied (Fig. 1). Once deposited into the ocean, metal concentrations from leaching behave hetereogeneously, depending on the chemical and biological interactions with ocean water (Fig. 5). Recent studies suggest important nutrient and toxicity effects from many aerosols (Fig. 6).

Although there has been substantial progress in understanding the role of aerosol metal leaching and the impact on the ocean biota, there are many open questions. We propose the following four main areas of research. First, continued improvement in measurement methods is required. For example, the development of more robust methods of observing deposition, both wet and dry, would facilitate understanding of aerosol metals: currently one can only infer deposition rates from atmospheric or ocean concentrations, or use ocean sediment traps, which are situated sometimes 1000 m below the surface and allow substantial downstream advection and processing within the ocean. Another example is the use of metal isotopes to provide unique constraints on the sources and sinks of metals in the atmosphere and ocean^52^. Secondly, there is a need to establish more long-term timeseries stations measuring aerosols, and their impact on the oceans. These studies would provide insights into source and sink processes, allow for detailed studies of the marine ecosystem dynamics influenced by episodic aerosol deposition, and provide a baseline for detecting changes over time driven by climate. Thirdly, model and data should be combined to provide budgets for ocean and atmospheric sources, including three-dimensional modeling and synthesis work. In addition, synthesizing modeling efforts are needed to better capture the impacts on marine ecosystems and biogeochemistry of aerosol trace metal deposition over a wide range of timescales, from a single deposition event to the large changes associated with glacial/interglacial cycles. Finally, much of the current work has focused on iron, but there are hints within the literature of the potential for other metals to be catalysts or inhibitors for ocean biogeochemistry, which should be explored. These studies need to include not just the nutrient effects from aerosols but also potential toxicity effects.

## Methods

### Atmospheric metal sources

Values in Fig. 3a and Table 1 were compiled using the sparse data available within the literature, and hence contain large uncertainties in their fluxes. The majority of anthropogenic metal emission values are the sum of all reported anthropogenic activities (major contributing sectors: fossil fuel combustion, metal production, cement production, and waste disposal)^132^. Al and Fe, not reported in that study, are given as the emission from anthropogenic combustion activity only, and hence likely represent an underestimate of their atmospheric emission fluxes. Emissions for Fe and Al were derived as follows: Fe emission values have been developed and compared to observations^15^ and Al emissions are then derived using a Al/Fe emission ratio of 4.6 calculated as the average of the anthropogenic emissions measured in one study^133^). Natural metal emissions are given separately for each major natural source: deserts and soils, vegetation fires, vegetation emissions (primary biological particles and if reported secondary volatile emissions), oceans (primary particles and if reported marine biogenic sources), and volcanoes. Dust emissions are calculated by multiplying the crustal emission of Al (tuned to observations)^35^ with a crustal composition ratio of metal/Al^134^. Dust emissions of Fe and Mn are then further tuned to observations^35^. For all other natural sources, the emissions of Al, Cu, Fe, Pb, and Zn are taken from^135^, except Fe emissions from fires which is from^15^, while emissions of Cd and Mn are from earlier estimates^127^. Emission estimates of Ti are the most limited within the literature and are reported here only for natural dust and fire sources. Dust Ti emissions were estimated using Ti/Fe ratio (0.0957) from West Africa wet deposition^136^. Fire Ti emissions were estimated using the emission factor (Ti/fine particulate matter = 0.043)^137^ multiplied by the same study’s estimate of emissions fine particulate matter, but this method represents a tropical fire emission contribution only.

Copper sources are slightly different between the values shown in the global summary^127^ versus those used for the three-dimensional modeling study^53^, as the latter did not include metal smelting because estimates for the location and production are not available, but these sources are thought to be important^127^.

### Methods for Box 2

There are two plausible ways to obtain an inverse relationship, where Fe is the amount of the Fe, sFe is soluble iron amounts, and PsFe is the percentage soluble amount of Fe.

First, we start by considering the case where the iron has a continental source, and is advected downwind, as well as being removed by wet and dry deposition. If we assume a constant lifetime due to deposition(*t*_d_), at an advection speed (*u*), we can obtain an *e*-folding length scale for the concentration of Fe (*D*_d_ = *u* × *t*_d_) and the iron amounts in equilibrium will evolve with distance downwind (*x*) from the initial plume (Fe(*x*_o_)):

This is a simple first-order loss term (dFe(*x*)):

1 $${\mathrm{dFe}}(x) = - \frac{{{\mathrm{d}x}}}{{{D}_{\mathrm{d}}}} \times {\mathrm{Fe}}(x),$$

which has the analytical solution:

2 $${\mathrm{Fe}}\left( x \right) = {\mathrm{Fe}}\left( 0 \right) \times {\mathrm{exp}}\left(\frac{{ - x}}{{{D}_{\mathrm{d}}}}\right).$$

In the case of combustion iron emission, we consider that there is no atmospheric processing, but rather two types of aerosols emitted: one that is coarse has a shorter lifetime, and lower solubilities, and another which is fine mode, higher solubility and longer lifetime (an example is combustion iron, but it could also be any highly processed fine mode aerosol).

We use Eq. (1) for the Fe(*x*) function as before, but now assume that all the solubility is in the fine particles (Fe_f_(*x*)), which have on average a solublity of *S*, and a lifetime of *t*_f_, or a spatial *e*-folding decay rate of *D*_f_ = *u* × *t*_f_, giving us a change in Fe_f_(*x*) (dFe_f_).

3 $${\mathrm{dFe}}_{\mathrm{f}} = - \frac{{{\mathrm{d}}x}}{{D_{\mathrm{f}}}} \times {\mathrm{Fe}}_{\mathrm{f}}\left( x \right) = > \ {\mathrm{dsFe}}\left( x \right) = - \frac{{{\mathrm{d}}x}}{{D_{\mathrm{f}}}} \times S \times {\mathrm{Fe}}_{\mathrm{f}}(x).$$

The analytical solution for Eq. (3) is:

4 $${\mathrm{sFe}}\left( x \right) = S \times {\mathrm{Fe}}_{\mathrm{f}}\left( 0 \right) \times {\mathrm{exp}}\left(\frac{{ - x}}{{D_{\mathrm{f}}}}\right).$$

Total iron is the sum of the fine and coarse mode, but assume that almost all the iron is in the coarse mode:

5 $${\mathrm{Fe}}\left( x \right) = {\mathrm{Fe}}\left( x \right) + {\mathrm{Fe}}_{\mathrm{f}}\left( x \right).$$

The percentage of soluble iron (PsFe(*x*)) is equal to:

6 $${\mathrm{PsFe}}\left( x \right) = \frac{{{\mathrm{sFe}}\left( x \right)}}{{{\mathrm{Fe}}\left( x \right)}} \times 100 = \frac{{S \times {\mathrm{Fe}}_{\mathrm{f}}\left( 0 \right) \times {\mathrm{exp}}(\frac{{ - x}}{{D_{\mathrm{f}}}})}}{{{\mathrm{Fe}}\left( 0 \right) \times {\mathrm{exp}}(\frac{{ - x}}{{D_{\mathrm{c}}}})}}.$$

If simple atmospheric processing of dust takes place, we can assume a first-order chemical reaction process converting insoluble iron (iFe) to soluble iron (sFe) at a rate *k* (where the timescale of conversion (*t*_c_) is 1/*k* or *k* = 1/*t*_c_). Similar to the above, we can convert the temporal scale to a spatial scale of chemical conversion (*D*_c_) using the mean winds (*u*): *D*_c_ = *u* × *t*_c_.

We can then calculate the amount of soluble iron produced when traveling between point *x* and point *x* + ∆*x*, farther downwind, and it will be a function of how much insoluble iron (iFe(*x*) = Fe(*x*) − sFe(*x*)) and the conversion distance scale (*D*_c_). In addition, there is a loss of the solubilized Fe dust with a spatial scale of *D*_d_, and we assume that 1% of the dust iron is soluble at the source on average.

7 $${\mathrm{dsFe}}\left( x \right) = + \frac{{{\mathrm{d}}x}}{{D_{\mathrm{c}}}} \times ({\mathrm{Fe}}\left( x \right) - {\mathrm{sFe}}\left( x \right)) - \frac{{{\mathrm{d}}x}}{{D_{\mathrm{d}}}} \times {\mathrm{sFe}}\left( x \right).$$

If the atmospheric processing of dust takes place preferentially by land-based emissions of acids (with a concentration of *P*(*x*); for example, sulfate or oxalate acids), we can add this into Eq. (7). We need to make the chemical reaction dependent on the acidity, which should be lost as the plume moves away from the source in an equation similar to Eq. (1), with a temporal scale (*t*_p_) and spatial scale (*D*_p_ = *u* × *t*_p_):

8 $${\rm d}{P}\left( x \right) = - \frac{{{\mathrm{d}}x}}{{D_{\mathrm{p}}}} \times {P}\left( x \right).$$

The analytical solution for Eq. (8) is:

9 $$P\left( x \right) = P\left( 0 \right) \times {\mathrm{exp}}\left(\frac{{ - x}}{{D_{\mathrm{p}}}}\right).$$

If we add a dependence on *P* into Eq. (7), we can obtain:

8 $${\mathrm{dsFe}}\left( x \right) = + \frac{{{\mathrm{d}}x}}{{D_{\mathrm{c}}}} \times \exp \left( { - \frac{x}{{D_{\mathrm{p}}}}} \right) \times ({\mathrm{Fe}}\left( x \right) - {\mathrm{sFe}}\left( x \right)) - \frac{{{\mathrm{d}}x}}{{D_{\mathrm{d}}}} \times {\mathrm{sFe}}\left( x \right).$$

The coefficients used in Box 2 are shown in Supplementary Table 1.

### Data availability

The datasets used in this paper are available by request to the corresponding author and available at www.geo.cornell.edu/eas/PeoplePlaces/Faculty/mahowald/dust.

## Electronic supplementary material

Supplementary Information
