# Supplementary material for: Aerosol trace metal leaching and impacts on marine microorganisms
Source: Nat Commun. 2018 Jul 5;9:2614. doi: 10.1038/s41467-018-04970-7 (PMC6033952; doi:10.1038/s41467-018-04970-7)
Supplement: Supplementary file 1 — Supplementary Information [file 41467_2018_4970_MOESM1_ESM.pdf]

Supplementary materials

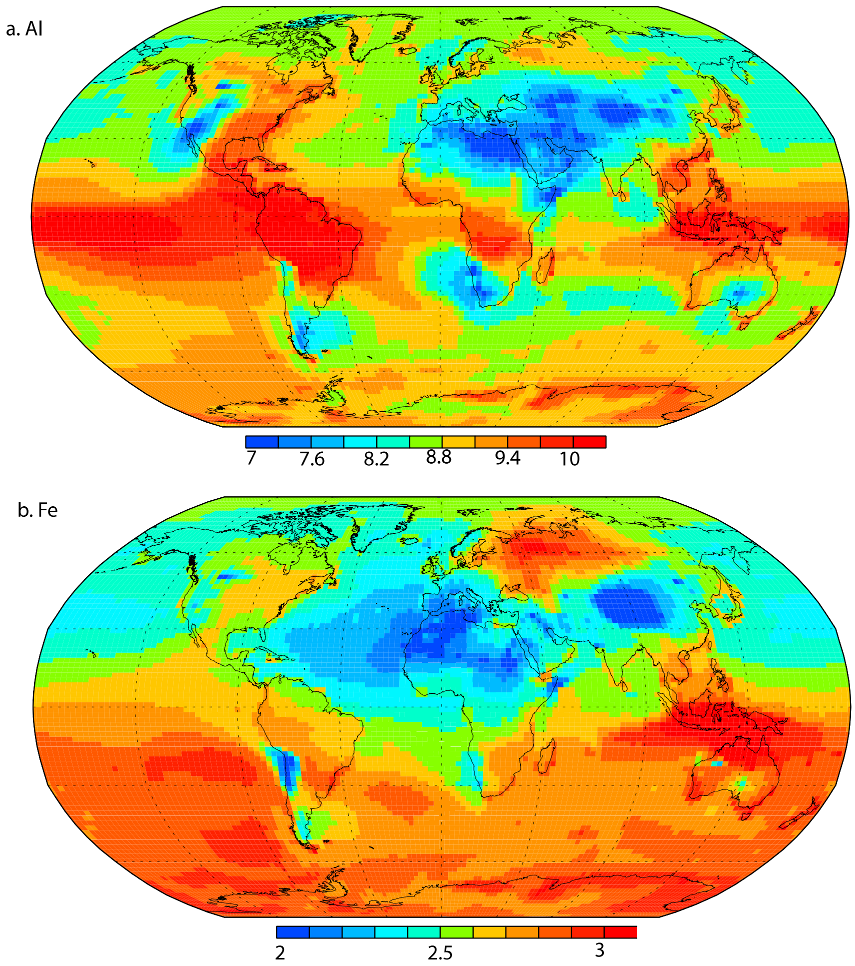

Supplementary Figure 1: Spatial distribution of the annual average ratio of mass fraction of elements in dust deposition in percentage units for Al (a) and Fe (b) from model estimates [4].

Supplementary Table 1: coefficients for simple plume models presented in Box 2.

Coefficient	Value
Dust lifetime (t_d)	2 days
Advection speed (u)	5 m/s
Dust spatial scale (D_d)	864km
Fine soluble Fe lifetime (t_f)	5 days
Fine soluble Fe spatial scale (D_f)	2160km
Atmospheric processing lifetime (t_C)	60 days
Atmospheric processing spatial scale (D_c)	25920 km
Pollutant lifetime (t_p)	5 days
Pollutant spatial scale (D_p)	2160km

Supplementary Table 2: Compilation of incubation bottle responses to additions

Study	Specific Location, Lat Long	Metals	Biological response	Cruises
Mahaffey et al. 2014[3]	North Atlantic transect<br><br>-Experiments on D326:<br>B2- 17.7°N, 24.3°W<br>B3- 12.3°N, 35.8°W<br>B4- 16.1°N, 30.6°W<br>-Experiments on D361<br>Zn1- 0.83°N, 25.23°W<br>Zn2- 15.81°N, 28.73°W	Zn	The addition of Sahara dust stimulated alkaline phosphatase activity. Alkaline phosphatase activity was found to be greater in the subtropical Atlantic Ocean than the the subtropical Pacific Ocean.	Cruises on the RRS Discovery<br>1. D326: (01/05/2008-02/05/2008)<br>2. D361: (02/07/2011-03/19/2011)
Browning et al. 2017[4]	North Atlantic transect	Fe	Fe limits APA in the low dust western Atlantic, but not in the high dust Eastern Atlantic.	Meteor 60 Transient Tracers Revisited expedition
Chien et al. 2016[5]	Carribbean Sea near Barbados<br><br>-Seawater collection (Feb 2012)<br>13°11.309′N, 59°38.267′W	Fe	High Fe (and N) in Sahara dust relative to P causes P limitation, which favors Prochlorococcus because it has low P cell quotas.	GEOTRACES cruise GA02

Mills et al. 2004[6]	North Atlantic transect<br><br>-Incubation locations in tropical Atlantic (October–November 2002)<br>Location 1: 10°N, 35°W<br>Location 2: 4°N 24°W<br>Location 3: 11°N 18°W	Fe	Nitrogen fixation is co-limited by N and P, and Saharan dust additions stimulated nitrogen fixation in the eastern North Atlantic by providing Fe and P.	Meteor 55 research cruise
Mackey et al. 2012[7]	Sargasso Sea<br><br>-Bermuda Atlantic Time-series Station: 31°40′N, 64°10′W<br>-Coastal experiment at Buoy 3A: 32°24.531′N, 64°44.769′W	Co, Mn, Ni	Aerosol-derived Co, Mn, and Ni supported growth of oceanic (but not coastal) Synechococcus. Aerosol copper additions did not cause [Cu'] to exceed toxicity levels, and the production of Cu-binding ligands may indicate a nutritive role for Cu.	NA
Duarte et al. 2006 [8]	Northeast Atlantic<br><br>(May-June 2003)-West African Coast:<br>-26.0°W and parallels 26.0° and 21.0°N<br>-Pulse experiment for surface seawater 25.99°N, 18.0°W<br>-A dose experiment 21.0°N, 23.0°W<br>-North transect (coastal ocean): 26.0°N<br>-South transect (coastal ocean): 21.1°N<br>-48-hour back trajectory for an open ocean site (21 N, 26 W) (May 29 2003)<br>-48-hour back trajectory for a coastal site (27 N, 15 W) (May 21 2003)	Fe	Aerosol addition led to a sevenfold increase in phytoplankton biomass and a tenfold increase in production over 4 days. Primary production was stimulated more than community respiration. The phytoplankton community shifted from picocyanobacteria-dominated to diatom-dominated. Bacterial abundance and production was less responsive.	Boat: R/V Hespe´rides

Chien et al. 2016[5]	North Atlantic<br><br>Incubations:<br>-E1: Lat: 10.8/Lon: 52.3<br>-E2: Lat: 8.3/Lon: 51.7<br>-E3: Lat: 11.6 Lon: 55.7	Fe	The diazotroph Trichodesmium shows luxury uptake of aerosol Fe. Uptake of Fe was proportional to the cellular P:Fe ratio and the amount of Fe leached from the aerosols.	NA
Maranon et al. 2010[9]	Atlantic Ocean transect<br><br>Experiment locations<br>1) 26.0°N, 34.8°W<br>2) 13.8°N, 28.4°W<br>3) 17.8°S, 29.0°W<br>4) 33.8°S, 38.4°W<br>5) 16.1°S, 29.0°W<br>6) 0.6°S, 29.0°W<br>7) 14.4°N, 29.0°W<br>8) 29.2°N, 28.3°W	unspecified	Dust additions increased bacterial production in ultraoligotrophic environments, and the effect was larger with increased oligotrophy. Primary production only increased in the least oligotrophic waters, and the effect was smaller as oligotrophy increased.	Tropical Atlantic (TRYNITROP) cruises
Moore et al. 2006[10]	North Atlantic<br><br>-48 hour incubation (nutrient enrichment bioassay experiments)<br>Location 1: 42.0°N, 42.0°W<br>Location 2: 35.0°N, 56.0°W<br>Location 3: 31.0°N, 27.0°W<br>-Bioassay experiments<br>1) 21°N 62°W<br>2) 28°N 64°W<br>3) 29°N 52°W<br>4) 31°N 27°W<br>5) 32°N 44°W<br>6) 36°N 24°W<br>7)35°N 56°W<br>8) 42°N 42°W	Fe	The episodic nature of Fe provided by dust deposition affects the dynamics of the spring bloom because Fe availability controls N consumption.	Meteor 60 Transient Tracers Revisited expedition (March & April 2004)

Langlois et al. 2012[11]	Tropical North Atlantic Ocean<br><br>Experiment site A: 4° N, 24° W<br>Experiment site B: 11° N, 18° W<br>Experiment site C: 6° N, 16° W	Fe	Sahara dust additions stimulated N2 fixation, and diazotroph (unicellular cyanobacteria groups A, B and C, as well as Gamma A proteobacteria) abundances. Dust additions had an increasingly negative effect on Synechococcus and Prochlorococcus abundances, but an increasingly positive effect on picoeukaryotes moving eastward. Laboratory culture experiments showed that Fe from Sahara dust promoted colony formation in Trichodesmium erythrium.	Meteor 55 research cruise
Guieu et al. 2014[12]; Wuttig et al. 2013[13]	Mediterranean Sea<br><br>-Guieu paper: Transect: Lat: 40.0°-45.0°N Lon: 5.0°-10.0°E<br><br>Wuttig paper:<br>-7 mesocosms distributed in the Bay of Elbo at 42.374◦ N, 8.554◦ E	Mn, Al, Fe	Changes in dissolved Fe following 2 sequential dust additions depended on biological ligand production; low ligand concentrations during the initial addition allowed Fe to scavenge onto particles, while higher biogenic ligand concentrations during the second addition allowed higher levels of dissolved Fe to persist. Al and Mn showed similar dissolution behaviors between the first and second additions, suggesting they were not affected by uptake or absorption into phytoplankton.	NA
Herut et al. 2005[14]	Mediterranean Sea<br><br>-SSW collected at (station 2CYC23) at 33°24.85 N; 32°18.49°E<br>-location of atmospheric aerosol samples:<br>1) Lat: 32°16.89 Long: 33°09.44<br>2) Lat: 32°59.81 Long: 33°14.87<br>3) Lat: 32°49.86 Lon: 33°15.86<br>4) Lat: 32°45.64 Lon: 33°23.73<br>5) Lat: 32°57.93 Lon: 33°24.13<br>6) Lat: 33°07.35 Lon: 33°18.83<br>7) Lat: 34°00.05 Lon: 32°39.98	unspecified	Saharan dust increased phytopigments and primary production. Synechococcus, prymnesiophytes, and ciliates increased in abundance, Prochlorococcus declined, and heterotrophic bacterial production increased	Boat: R.V. Aegaeo

Lekunberri et al. 2010[15]	Mediterranean Sea<br><br>-Experiments occurred in The Blanes Bay Microbial Observatory, 41°40′0″N, 2°48′0″E<br>-Saharan dust collected in Villefranche-sur-Mer, France (43°42′18″N, 7°18′45″E)	unspecified	Low dust (0.05g/L) addition favored heterotrophy, while high dust (0.5g/L) or P additions favored autotrophy.	NA
Bonnet et al. 2005[16]	Mediterranean Sea<br><br>DYFAMED site (43°25'N, 78°29'E)	Fe	Fe and P from aerosols stimulated primary production, and dust additions favored eukaryotic phytoplankton.	Boat: R/V Te´thys II
Romero et al. 2011[17]	Mediterranean Sea Blanes Bay<br><br>-Aerosol collection in Nice, France: 43°42'10" N, 7°16'9" E<br>-Water collection at Blanes Bay Microbial Observator: (41°40' 0"N, 2°48'0"E)	unspecified	Low dust (0.05g/L) or P addition favored phototrophic nanoflagellates, while high dust (0.5g/L) additions increased bacterial abundance.	NA
Ridame et al. 2011[18]	Mediterranean Sea<br><br>Incubations<br>Station A (Western Basin) : 39◦110 N, 5◦350 E<br>Station B (Ionian Sea) : 34◦130 N, 18◦450 E<br>Station C (Levantine Sea): 33◦63 N, 32◦65 E	Fe and others	Saharan dust input relieved nutrient limitation of N2 fixation and the response was geographically variable. N2 fixation was P limited at many sites. A strong response to dust at one site that was not P limited suggested that one or more other nutrients increased N2 fixation rates.	European BOUM cruise
Ridame et al. 2013[19]	Northwestern Mediterranean Sea<br><br>-Experiments conducted at Natural Preservation Area of Scandola (8.554◦ E, 42.374◦ N)	Fe	Wet and dry Sahara dust deposition induced a rapid increase in N2 fixation. N2 fixation was not inhibited by N in the dust.	The DUNE project

Mackey et al. 2017[20]	East China Sea<br><br>-Site 1 for seawater collection/incubation: (30◦43′04″N, 122◦39′58″E)<br>-Site 2 for seawater collection/incubation: (30◦38′12.2″N, 122◦56′8.51″ E)<br>-Aerosol collection: (30.86◦N, 122.67◦E)<br>-Map showing HABs in Eastern China: Lat: 28◦-32◦N Lon: 120◦-128E	Al, Cd, Co, Cu, Fe, Mn, Ni, Pb ,and Zn	High Fe (and N) in Asia aerosols induces P limitation and favors dinoflagellates that benefit from the high Fe and low N:P ratios. Synechococcus was weakly correlated with high Cu, low Fe, and low N:P ratios. Increased anthropogenic aerosol emissions over the past three decades may contribute to the observed increase in harmful algal blooms in this region.	NA
Guo et al. 2012 [33]	South China Sea<br><br>-Range of experiments:<br>Lat: 16°-24° N; Lon: 110°-120° E<br>-Bioassay experiments (type of location/depth):<br>1)coastal/17 m<br>2)continental shelf/137 m<br>3)oceanic/ 3844 m<br>4)continental slope/ 854 m<br>5) oceanic/ 3340 m	Fe and mixed metals	High aerosol loading increased total phytoplankton biomass and photosynthetic efficiency, and favored microphytoplanton like diatoms over picoplankton. Possible Cu toxicity was observed for Synechococcus.	Aerosol samples were collected locally on the rooftop of the Academic Building at the Hong Kong University of Science and Technology (HKUST)
Bonnet et al. 2008[21]	Southeast Pacific<br><br>-Experiment stations<br>Station 1 (HNL): 9◦04'S, 136◦97'W<br>Station 2 (GYR): 26◦04'S, 114◦02'W<br>Station 3 (EGY): 31◦89'S, 91◦39'W	Fe	Dust additions did not stimulate N2 fixation despite the ultra low dissolved Fe concentrations at the sites. Productivity was only Fe limited at the edge of the gyre.	BIOSOPE cruise
Maki et al. 2011[22]	seashore of Kii Peninsula, at a distance of 150 km 33°29′N, 136°59′E	unspecified	Asian dust particles from the Mt. Tateyama snow layers and the surface sand of Loess Plateau stimulated Gram-positive and alpha proteobacterial growth. No enrichment of chlorophyll resulted from dust additions.	SE0926 cruise of T/S Seisui Maru (Mie University)
Liu et al. 2013[23]	Yellow Sea<br><br>-Incubation site A2: 36°04.116′N, 123°11.082′E	Fe	Asian dust stimulated chlorophyll at the highest dose (20mg/L) only. Rainwater stimulated chlorophyll and the abundance of microphytoplankton, had no effect on	Cruise on the R/V Dongfanghong 2 (18 March to 4 April 2011)

			picoplankton, and inhibited nanoplankton.	
Mélançon et al. 2016[24]	Northeast subarctic Pacific HNLC<br><br>-Water collection: 50◦ N, 145◦ W (10m depth)	Fe	Aerosol Fe stimulated chlorophyll and particulate organic carbon production, but the response was reduced under low pH conditions. Dust additions had a fertilizing effect mainly on diatoms and cyanobacteria.	Cruise on the Canadian Coast Guard Ship John P. Tully
Hamme et al. 2010[25]	subarctic northeast Pacific<br><br>-Region tested for chlorophyll 48–56°N, 136–150°W<br>-Location of Kasatochi volcano (52.2°N, 175.5°W)<br>-Station P (sampling) 145°W	Fe	Fe in volcanic ash from the Aleutian Islands induced a large diatom bloom several days following deposition.	Two cruises (no names included) August 2008
Foster et al. 2009 [42]	Gulf of Aqaba<br><br>-Seawater collection Station A: 29°27.815'N, 34°55.830'E<br>Station B: 29°22.608'N, 34°53.894'E	unspecified	Highest N2 fixation rates were observed in the dust addition treatment.	NA
Wang et al. 2017[26]	East China Sea<br><br>-Sampling station: 30.86°N, 122.67°E<br>-Sampling station: 30.85°N, 123.42°E	Fe, Cu	The ratio of Fe/Cu in aerosols influenced the toxicity of Cu on phytoplankton. Higher Fe/Cu ratios mitigated the toxic effect and were correlated with higher chlorophyll levels in offshore waters. Fe/Ce ratios did not affect chlorophyll concentrations in coastal regions where rivers and upwelling dominated the biogeochemistry.	No cruise name but sampling station was Huaniao Island.
Paytan et al. 2009 [27]	Gulf of Aqaba	Cu	Aerosol Cu caused toxicity for Synechococcus and picoeukaryotes, but not for Prochlorococcus	NA

Mackey et al. 2007 [28]	Gulf of Aqaba<br><br>-Station A (Israeli waters): 29°28'N, 34°55'E<br>-Station B (Jordanian waters): 29°22'N,34°53'E	unspecified	Dust additions stimulated chlorophyll a, but did not affect alkaline phosphatase activity among nano and microplankton	NA
Rubin et al. 2011[29]	Gulf of Aqaba	Fe	Trichodesmium accelerates Fe dissolution from dust via cell surface processes that involve efficient trapping and movement of the particles within the colony.	NA

Supplementary references

1. Moore, J. K., Lindsay, K., Doney, S., Long, M. & Misumi, K. Marine Ecosystem Dynamcis and Biogeochemical Cycling in the Community Earth System Model1 (BGC): Comparison of hte 1990s with the 2090s under the RCP4.5 and RCP8.5 scenarios. *J. Clim.* **26,** 9291–9312, doi:10.1175/jcli–d–12–00566.1 (2013).
2. Zhang, Y. *et al.* Modeling the global emission, transport and deposition of trace elements associated with mineral dust. *Biogeosciences* **12,** (2015).
3. Mahaffey, C., Reynolds, S., Davis, C. E. & Lohan, M. C. Alkaline phosphatase activity in the subtropical ocean: insights from nutrient, dust and trace metal addition experiments. *Front. Mar. Sci.* **1,** (2014).
4. Browning, J. *et al.* Iron limitation of microbial phosphorus acquisition in the tropical North Atlantic. *Nat. Commun.* **in press,** (2017).
5. Chien, C.-T. *et al.* Effects of African dust deposition on phytoplankton in the western tropical Atlantic Ocean off Barbados. *Global Biogeochem. Cycles* **30,** (2016).
6. Mills, M. M., Ridame, C., Davey, M., La Roche, J. & Geider, R. J. Iron and phosphorus co-limit nitrogen fixation in the eastern tropical North Atlantic. *Nature* **429,** 292–294 (2004).
7. Mackey, K. R. M., Mioni, C. E., Ryan, J. P. & Paytan, A. Phosphorus Cycling in the Red Tide Incubator Region of Monterey Bay in Response to Upwelling. *Front. Microbiol.* **3,** (2012).
8. Duarte, C. M. *et al.* Aerosol inputs enhance new production in the subtropical northeast Atlantic. *J. Geophys. Res. Biogeosciences* **111,** G04006 (2006).
9. Marañon, E. *et al.* Degree of oligotrophy controls the response of microbial plankton to {Saharan} dust. *Limnol. Oceanogr.* **55,** 2339–2352 (2010).
10. Moore, C. M. *et al.* Iron limits primary productivity during spring bloom development in the central {North} {Atlantic}. *Glob. Chang. Biol.* **12,** 626–634 (2006).
11. Langlois, R., Mills, M., Ridame, C., Croot, P. & LaRoche, J. Diazotrophic bacteria respond to Saharan dust additions. *Mar. Ecol. Prog. Ser.* **470,** 1–14 (2012).
12. Guieu, C., Dulac, F., Ridame, C. & Pondaven, P. Introduction to project DUNE, a DUst experiment in a low Nutrient, low chlorophyll Ecosystem. *Biogeosciences* **11,** 425–442 (2014).
13. Wuttig, K. *et al.* Impacts of dust deposition on dissolved trace metal concentrations (Mn, Al and Fe) during a mesocosm experiment - ProQuest. *Biogeosciences* **10,** 2583–2600 (2013).

14. Herut, B. *et al.* Response of East Mediterranean surface water to Saharan dust: On-board microcosm experiment and field observations. *Deep Sea Res. Part II Top. Stud. Oceanogr.* **52,** 3024–3040 (2005).
15. Lekunberri, I. *et al.* Effects of a dust deposition event on coastal marine microbial abundance and activity, bacterial community structure and ecosystem function {\textbar} {Journal} of {Plankton} {Research} {\textbar} {Oxford} {Academic}. *J. Plankton Res.* **32,** 381–396 (2010).
16. Bonnet, S., Guieu, C., Chiaverini, J., Ras, J. & Stock, A. Effect of atmospheric nutrients on the autotrophic communities in a low nutrient, low chlorophyll system. *Limnol. Oceanogr.* **50,** 1810–1819 (2005).
17. Romero, E. *et al.* Coastal Mediterranean plankton stimulation dynamics through a dust storm event: {An} experimental simulation. *Estuar. Coast. Shelf Sci.* **93,** 27–39 (2011).
18. Ridame, C. *et al.* Nutrient control of Nfixation in the oligotrophic Mediterranean Sea and the impact of Saharan dust events. *Biogeosciences* **8,** 2773–2783 (2011).
19. Ridame, C., Guieu, C. & L'Helguen, S. Strong stimulation of N2 fixation in oligotrophic Mediterranean Sea: results from dust addition in large in situ mesocosms. *Biogeosciences* **10,** 7333–7346 (2013).
20. Mackey, K. R. M. *et al.* Atmospheric and Fluvial Nutrients Fuel Algal Blooms in the East China Sea. *Front. Mar. Sci.* **4,** (2017).
21. Bonnet, S. *et al.* Nutrient limitation of primary productivity in the {Southeast} {Pacific} ({BIOSOPE} cruise). *Biogeosciences* **5,** 215–225 (2008).
22. Maki, T. *et al.* Effects of Asian Dust (KOSA) Deposition Event on Bacterial and Microalgal Communities in the Pacific Ocean. *Asian J. Atmos. Environ.* **5,** 157–163 (2011).
23. Liu, Y., Zhang, T. R., Shi, J. H., Gao, H. W. & Yao, X. H. Responses of chlorophyll a to added nutrients, {Asian} dust, and rainwater in an oligotrophic zone of the {Yellow} {Sea}: {Implications} for promotion and inhibition effects in an incubation experiment. *J. Geophys. Res. Biogeosciences* **118,** 2013JG002329 (2013).
24. Mélançon, J. *et al.* Impact of ocean acidification on phytoplankton assemblage, growth, and {DMS} production following {Fe}-dust additions in the {NE} {Pacific} high-nutrient, low-chlorophyll waters. *Biogeosciences* **13,** 1677–1692 (2016).
25. Hamme, R. C. *et al.* Volcanic ash fuels anomalous plankton bloom in subarctic northeast {Pacific}. *Geophys. Res. Lett.* **37,** L19604 (2010).
26. Wang, F. J. *et al.* Combined effects of iron and copper from atmospheric dry deposition on ocean productivity. *Geophys. Res. Lett.* **44,** 2016GL072349 (2017).
27. Paytan, A. *et al.* Toxicity of atmospheric aerosols on marine phytoplankton. *Proc. Natl. Acad. Sci.* **106,** 4601–4605 (2009).
28. Mackey, K. R. M. *et al.* Phosphorus availability, phytoplankton community dynamics, and taxon-specific phosphorus status in the Gulf of Aqaba, Red Sea. *Limnol. Oceanogr.* **52,** 873–885 (2007).
29. Rubin, M., Berman-Frank, I. & Shaked, Y. Dust- and mineral-iron utilization by the marine dinitrogen-fixer Trichomesmium. *Nat. Geosci.* **4,** 529–534 (2011).
